# The burden of injury in China, 1990–2017: findings from the Global Burden of Disease Study 2017

**DOI:** 10.1016/S2468-2667(19)30125-2

**Published:** 2019-09-04

**Authors:** Duan Leilei, Ye Pengpeng, Juanita A Haagsma, Jin Ye, Wang Yuan, Er Yuliang, Deng Xiao, Gao Xin, Ji Cuirong, Wang Linhong, Marlena S Bannick, W Cliff Mountjoy-Venning, Caitlin N Hawley, Zichen Liu, Mari Smith, Spencer L James, Theo Vos, Christopher J L Murray

**Affiliations:** a Division of Injury Prevention and Mental Health, National Center for Chronic and Noncommunicable Disease Control and Prevention, Chinese Center for Disease Control and Prevention, Beijing, China; b Center for Medical Decision Sciences, Department of Public Health, Erasmus Medical Center, Rotterdam, Netherlands; c Institute for Health Metrics and Evaluation, University of Washington, Seattle, WA, USA

## Abstract

**Background:**

A comprehensive evaluation of the burden of injury is an important foundation for selecting and formulating strategies of injury prevention. We present results from the Global Burden of Diseases, Injuries, and Risk Factors Study (GBD) 2017 of non-fatal and fatal outcomes of injury at the national and subnational level, and the changes in burden for key causes of injury over time in China.

**Methods:**

Using the methods and results from GBD 2017, we describe the burden of total injury and the key causes of injury based on the rates of incidence, cause-specific mortality, and disability-adjusted life years (DALYs) in China estimated using DisMod-MR 2.1. We additionally evaluated these results at the provincial level for the 34 subnational locations of China in 2017, measured the change of injury burden from 1990 to 2017, and compared age-standardised DALYs due to injuries at the provincial level against the expected rates based on the Socio-demographic Index (SDI), a composite measure of development of income per capita, years of education, and total fertility rate.

**Findings:**

In 2017, in China, there were 77·1 million (95% uncertainty interval [UI] 72·5–81·6) new cases of injury severe enough to warrant health care and 733 517 deaths (681 254–767 006) due to injuries. Injuries accounted for 7·0% (95% UI 6·6–7·2) of total deaths and 10·0% (9·5–10·5) of all-cause DALYs in China. In 2017, there was a three-times variation in age-standardised injury DALY rates between provinces of China, with the lowest value in Macao and the highest in Yunnan. Between 1990 and 2017, the age-standardised incidence rate of all injuries increased by 50·6% (95% UI 46·6–54·6) in China, whereas the age-standardised mortality and DALY rates decreased by 44·3% (41·1–48·9) and 48·1% (44·6–51·8), respectively. Between 1990 and 2017, all provinces of China experienced a substantial decline in DALY rates from all injuries ranging from 16·3% (3·1–28·6) in Shanghai and 60·4% (53·7–66·1) in Jiangxi. Age-standardised DALY rates for drowning; injuries from fire, heat and hot substances; adverse effects of medical treatments; animal contact; environmental heat and cold exposure; self-harm; and executions and police conflict each declined by more than 60% between 1990 and 2017.

**Interpretation:**

Between 1990 and 2017, China experienced a decrease in the age-standardised DALY and mortality rates due to injury, despite an increase in the age-standardised incidence rate. These trends occurred in all provinces. The divergent trends in terms of incidence and mortality indicate that with rapid sociodemographic improvements, the case fatality of injuries has declined, which could be attributed to an improving health-care system but also to a decreasing severity of injuries over this time period.

**Funding:**

Bill & Melinda Gates Foundation.

## Introduction

The burden of injury is a universal public health challenge that afflicts every country in the world, with cases distributed across all ages and both sexes. In China, rapid economic development, urbanisation, motorisation, ageing, and environmental and lifestyle changes over the past three decades have led to injury being reported as the fifth leading cause of death after malignant neoplasms, cerebrovascular disease, respiratory diseases, and heart attacks.^1^ Injuries cause an annual loss of 12·6 million years of productivity in China, more than for each of the respiratory, cardiovascular, infectious, and neoplastic disease groups.^2^ The direct medical cost of injury in China has been estimated to be about 65 billion Ren Min Bi or US$10 billion per year.^3^ Consequently, injury is seen as a growing public health concern in China that could cause considerable economic expenditure.

**Research in context**

**Evidence before this study**

The burden of injuries in China is an important topic for public health planning and for contextualising economic trends and health interventions. We searched Ovid MEDLINE for articles published up to April 10, 2019, using compound search terms (appendix p 5), with language restricted to English and Chinese. Previous research focused on the burden of injuries in China has been limited in scope in terms of including all injuries, estimating for all demographic groups and provinces, and modelling for a span of years. Past iterations of the Global Burden of Diseases, Injuries, and Risk Factors Study (GBD) have gradually increased the level of detail in terms of causes, locations, and demographic groups receiving estimations. GBD 2017 included subnational estimation for provinces of China for 30 mutually exclusive causes of injury. Given the rapid expansion of the economy in China over the past three decades, there was considerable interest in understanding the ramifications that such socioeconomic changes have had on the burden of injuries, given that the morbidity and mortality of certain injuries is thought to be affected by economic development. Past research focusing on the interaction between economic development and injury burden has revealed several factors that could affect injury incidence and mortality, such as the expansion of road systems, changing social patterns with migration of younger generations, and increasing educational access and attainment. However, to date, no studies have systematically measured the burden of injury in terms of morbidity and mortality at the province level in China over several years and for all age groups and both sexes.

**Added value of this study**

This study measures the burden of injury at both the national and provincial level in China in terms of incidence, cause-specific mortality, and disability-adjusted life years (DALYs). Additionally, it reports these measures for each of 30 mutually exclusive injury causes (except for select injuries that do not lead to death) for every age group and both sexes for the time period from 1990 to 2017. The results of the study are then interpreted on the basis of relevant historical and policy-related events from the past three decades that might have contributed to an evolving injury burden. The provision of such societal and historical context could help to explain the reasons for the dynamic trends observed for fatal and non-fatal results and how these trends might vary depending on the province.

**Implications of all the available evidence**

This study shows that although the incidence of injuries in China has increased with the expansion of the economy, the rates of cause-specific mortality and DALYs have declined. These observations suggest that the rapid socioeconomic development in China might have increased the risk of injury, but that improvements in health care or decreases in injury severity have led to large improvements in mortality and overall health loss. This study represents an important geographical research focus within the greater GBD project that might help to inform policy not only in China but also in other parts of the world that are experiencing economic growth and a shifting burden of injury.

Research and international cooperation in the fields of surveillance, intervention, education, promotion, and advocacy that target different types of injuries and high-risk populations has led to policies, regulations, and laws being introduced and implemented in various fields of injury prevention and control in China.^4^, ^5^ However, there is still a lack of integrated data on the burden of injury across all injuries at both national and subnational levels. It is also increasingly of interest to measure the change in key injuries over time and to identify priority areas for injury prevention.

The goal of the Global Burden of Diseases, Injuries, and Risk Factors Study (GBD) 2017 was to incorporate all available epidemiological data using a coherent measurement framework, standardised estimation methods, and transparent data sources to enable comparisons of health loss over time and across causes, age groups, sexes, and locations.^6^, ^7^, ^8^, ^9^, ^10^, ^11^ Here, we aim to provide an overview of the burden of injury in China in 2017, to measure the change in this burden between 1990 and 2017, and to explore the underlying factors influencing this change.

## Methods

### Overview

In this analysis, we describe the burden of total injury and the key causes of injury based on the rates of incidence, cause-specific mortality, and disability-adjusted life years (DALYs) in China. We present a detailed description of the top four causes of injury in terms of age-standardised DALY rates in 2017. The geographical units of analysis include 31 provinces in mainland China, Hong Kong special administrative region, Macao special administrative region, and Taiwan; all 34 locations will be referred to as provinces in the rest of the Article.

The GBD 2017 study design and methods specific to injury estimation have been described in extensive detail in existing GBD literature.^6^, ^10^ An overview of GBD methods is provided in the appendix (pp 1–4). Here, we summarise key features of these methods.

This study complied with the Guidelines for Accurate and Transparent Health Estimates Reporting recommendations.^12^

### Case definitions

In GBD 2017, there are 30 mutually exclusive and collectively exhaustive external cause of injury categories, with each category further divided among 47 mutually exclusive nature of injury categories. In this study, we report an aggregated version of this cause list. All subtypes of road injuries, for example, were combined into one road injuries cause. The definition of injury incidence and death was based on the International Classification of Diseases (ICD): codes E000–E999 in ICD-9 and chapters V to Y in ICD-10. ICD-9 codes 800–999 and ICD-10 chapters S and T were used for estimation of injury morbidity. Specific coding by injury is provided in the GBD 2017 literature.^6^, ^10^

### Mortality

For cause-specific mortality estimation, all data sources were first mapped to the GBD cause list of diseases and injuries. Second, garbage codes or ill-defined causes of deaths were redistributed to causes within the GBD cause hierarchy. Third, the GBD Cause of Death Ensemble model method was used to generate estimates by age, sex, location, year, and cause. Five main data sources from China were used across all causes of death: the China disease surveillance points (DSP) system, vital registration collected by the Chinese Center for Disease Control and Prevention (CDC), cancer registry data, and medical certification of causes of death for Macao and Hong Kong (the only data sources for Macao and Hong Kong).^6^ Years of life lost (YLLs) were estimated as the number of deaths from each cause in each age group multiplied by the remaining life expectancy at the age of death.

### Incidence, prevalence, and years lived with disability

Measuring non-fatal health outcomes required combining two injury classification approaches. Injuries can be defined by cause and nature, where the cause of injury is the accident that led to a particular nature of injury. For example, for a fall that led to a traumatic brain injury, the fall would be considered the cause of injury and traumatic brain injury would be considered the nature of injury. In this study, we reported estimates by cause of injury.

The injuries estimation process used DisMod-MR 2.1, a Bayesian meta-regression tool, to produce injury incidence for different causes by location, year, age, and sex. The incidence of injury warranting inpatient admission and other types of care (eg, outpatient care including emergency department visits) was estimated separately. Second, the nature-of-injury category resulting in the largest burden for patients experiencing multiple injuries was selected through a hierarchy constructed according to patient-reported outcome measurement data as described in the GBD 2017 methods.^10^ Third, the proportions of incident cases in each combined cause-nature-of-injury category were estimated and then applied to cause of injury incidence obtained from the first step to estimate incidence of inpatient and outpatient injuries by cause and nature of injury. Fourth, short-term disability and average duration for each nature of injury category were estimated and short-term prevalence was derived from the multiplication of incidence and duration. Fifth, the proportion of cases with permanent disability for each nature of injury category was estimated and the ordinary differential equation solver used in DisMod-MR 2.1 was used to estimate the long-term prevalence for each cause-nature of injury from the incidence and long-term mortality risk in cases with long-term disability. Additional data sources used for the estimation of non-fatal health outcomes were the Chinese National Injury Surveillance System (NISS) and the China Zhuhai injury patient follow-up study.^13^, ^14^

Years lived with disability (YLDs) were then calculated by multiplying prevalence by a disability weight reflecting the magnitude of the health loss associated with an outcome after correction for comorbidity with other non-fatal diseases.

### DALYs

DALYs are the sum of YLLs and YLDs as estimated in GBD 2017 for each cause, location, age group, sex, and year.^7^ A DALY essentially represents 1 lost year of healthy life. The sum of DALYs in a population approximates the gap between the population's present health status and an ideal scenario where the entire population has lived to an advanced age, free of disease.

### Socio-demographic Index

The Socio-demographic Index (SDI) is a summary measure ranging from 0 to 1 that estimates the position of each GBD location on a spectrum of socioeconomic development. SDI is calculated using an equal weighting of lag-distributed income per capita, average years of education in the population older than 15 years of age, and total fertility rate in women younger than 25 years of age. The SDI for China in 2017 was 0·71, whereas at the province level, SDI varied from 0·47 to 0·86. Globally, this placed China in the high-middle SDI quintile, with province-level SDI values varying from low-middle to high quintiles on a global scale.

### Uncertainty analysis

We applied the same technique for propagating uncertainty as used elsewhere in the GBD study design. The distribution of every step in the computation process was stored in 1000 draws that were used for every step in the computational process. The distributions were determined from the sampling error from data inputs, the uncertainty of the coefficients from DisMod-MR 2.1, and the uncertainty of severity distributions and disability weights. Final estimates were computed using the mean estimate across 1000 draws, and 95% uncertainty intervals (UIs) for each average were defined by the 25th and 975th values of the ordered 1000 estimate values.

### Role of the funding source

The funder of the study had no role in study design, data collection, data analysis, data interpretation, or writing of the report. All authors had access to the data in the study and had final responsibility to submit for publication.

## Results

In 2017, in China, there were 77·1 million (95% UI 72·5–81·6) new cases of injury severe enough to warrant health care and 733 517 deaths (681 254–767 006) due to injuries. Injuries accounted for 7·0% (95% UI 6·6–7·2) of total deaths and 10·0% (9·5–10·5) of all-cause DALYs in China. Age-standardised rates of incidence, mortality, and DALYs for all injuries in 2017 were 5112 new cases (95% UI 4827–5410) per 100 000, 45·9 deaths (42·9–47·8) per 100 000, and 2473 DALYs (2298–2679) per 100 000 (Table 1, Table 2).

**Table 1:** Age-standardised mortality and incidence rates in 2017 and percentage change from 1990 to 2017 by cause of injury in China

		**Deaths**		**Incidence**	
		2017 age-standardised rates per 100 000	Percentage change in age-standardised rates, 1990–2017	2017 age-standardised rates per 100 000	Percentage change in age-standardised rates, 1990–2017
**All injuries**		**45·9 (42·9 to 47·8)**	**−44·3% (−48·9 to −41·1)**	**5112 (4827 to 5410)**	**50·6% (46·6 to 54·6)**
	**Transport injuries**	**16·1 (15·4 to 16·8)**	**−23·9% (−32·7 to −17·6)**	**1099 (973 to 1239)**	**82·2% (73·7 to 89·9)**
	Road injuries	15·6 (14·8 to 16·2)	−21·8% (−30·7 to −14·9)	940 (810 to 1075)	113·9% (103·7 to 123·6)
	Other transport injuries	0·6 (0·5 to 0·6)	−55·9% (−63·4 to −48·0)	160 (130 to 197)	−2·8% (−8·5 to 4·5)
	**Unintentional injuries**	**21·6 (18·9 to 22·9)**	**−40·9% (−50·9 to −35·4)**	**3635 (3391 to 3888)**	**55·5% (50·7 to 60·3)**
	Falls	8·6 (6·7 to 9·5)	12·8% (−23·9 to 33·4)	1477 (1288 to 1691)	113·2% (104·4 to 123·6)
	Drowning	5·1 (4·9 to 5·3)	−65·3% (−67·5 to −62·9)	8 (7 to 10)	−17·4% (−23·8 to −10·2)
	Fire, heat, and hot substances	0·7 (0·6 to 0·8)	−64·9% (−68·7 to −51·9)	108 (87 to 133)	46·0% (36·7 to 56·2)
	Poisonings	1·7 (0·9 to 2·0)	−20·8% (−66·3 to 15·3)	56 (46 to 69)	48·2% (36·5 to 60·6)
	Exposure to mechanical forces	1·9 (1·3 to 2·1)	−24·4% (−65·0 to −8·6)	797 (683 to 931)	85·5% (76·7 to 95·4)
	Adverse effects of medical treatment	0·4 (0·4 to 0·6)	−66·5% (−73·7 to −51·5)	506 (430 to 586)	64·2% (56·2 to 72·4)
	Animal contact	0·2 (0·1 to 0·2)	−77·7% (−82·3 to −50·7)	232 (196 to 273)	−33·3% (−36·3 to −30·0)
	Foreign body	1·4 (1·2 to 1·5)	−50·8% (−61·6 to −44·0)	185 (162 to 208)	6·2% (0·1 to 12·9)
	Environmental heat and cold exposure	0·3 (0·2 to 0·4)	−75·8% (−79·1 to −63·5)	104 (86 to 125)	−8·8% (−13·9 to −3·6)
	Exposure to forces of nature	0·0 (0·0 to 0·0)	−67·4% (−67·4 to −67·4)	2 (2 to 2)	−67·4% (−67·4 to −67·4)
	Other unintentional injuries	1·2 (1·1 to 1·2)	−2·7% (−8·9 to 5·9)	161 (132 to 196)	11·5% (5·7 to 17·8)
	**Self-harm and interpersonal violence**	**8·2 (7·8 to 9·1)**	**−66·7% (−69·9 to −58·1)**	**376 (321 to 440)**	**−16·6% (−22·7 to −9·0)**
	Self-harm	7·2 (6·8 to 7·9)	−65·6% (−68·7 to −57·6)	29 (24 to 35)	−39·4% (−45·1 to −33·8)
	Interpersonal violence	1·0 (0·8 to 1·3)	−71·7% (−76·5 to −59·7)	341 (287 to 403)	−4·5% (−10·4 to 1·4)
	Conflict and terrorism	0·0 (0·0 to 0·0)	−100·0% (−100·0 to −100·0)	0 (0 to 0)	−100·0% (−100·0 to −100·0)
	Executions and police conflict	0·0 (0·0 to 0·1)	−86·8% (−89·0 to −55·3)	6 (5 to 8)	−87·1% (−89·5 to −56·3)

Data in parentheses are 95% uncertainty intervals.

**Table 2:** Age-standardised YLL, YLD, and DALY rates in 2017 and percentage change from 1990 to 2017 by cause of injury in China

		**YLLs**		**YLDs**		**DALYs**	
		2017 age-standardised rates per 1000 000	Percentage change in age-standardised rates, 1990–2017	2017 age-standardised rates per 100 000	Percentage change in age-standardised rates, 1990–2017	2017 age-standardised rates per 100 000	Percentage change in age-standardised rates, 1990–2017
**All injuries**		**1893 (1798 to 1963)**	**−55·8% (−58·8 to −53·4)**	**580 (430 to 758)**	**21·2% (14·9 to 27·9)**	**2473 (2298 to 2679)**	**−48·1% (−51·8 to −44·6)**
	**Transport injuries**	**724 (693 to 754)**	**−35·0% (−42·0 to −29·8)**	**198 (141 to 268)**	**32·1% (24·5 to 40·0)**	**923 (855 to 996)**	**−27·0% (−33·9 to −21·3)**
	Road injuries	699 (669 to 728)	−33·3% (−40·4 to −27·7)	155 (111 to 209)	70·2% (62·1 to 79·1)	854 (798 to 914)	−25·0% (−32·5 to −18·8)
	Other transport injuries	25 (23 to 26)	−62·0% (−67·9 to −53·7)	44 (31 to 60)	−26·3% (−30·5 to −21·6)	69 (56 to 84)	−45·1% (−51·1 to −38·5)
	**Unintentional injuries**	**872 (775 to 921)**	**−58·2% (−64·5 to −54·3)**	**280 (196 to 384)**	**27·8% (19·7 to 36·2)**	**1152 (1017 to 1279)**	**−50·1% (−56·8 to −44·9)**
	Falls	209 (157 to 234)	−18·9% (−50·1 to −2·3)	154 (109 to 211)	67·6% (57·0 to 78·5)	363 (288 to 433)	3·8% (−25·6 to 18·6)
	Drowning	297 (283 to 312)	−71·4% (−73·4 to −69·2)	3 (2 to 3)	−34·7% (−39·9 to −29·5)	300 (285 to 314)	−71·3% (−73·3 to −69·1)
	Fire, heat, and hot substances	23 (19 to 27)	−74·8% (−78·6 to −59·2)	25 (15 to 39)	−18·0% (−35·6 to −0·7)	48 (38 to 63)	−60·7% (−68·7 to −44·4)
	Poisonings	72 (40 to 86)	−38·0% (−73·0 to −9·6)	3 (2 to 4)	50·4% (39·3 to 60·9)	74 (43 to 89)	−36·6% (−71·6 to −8·2)
	Exposure to mechanical forces	91 (64 to 99)	−35·0% (−68·9 to −23·1)	45 (31 to 66)	49·0% (37·6 to 60·8)	136 (107 to 159)	−20·1% (−53·1 to −6·9)
	Adverse effects of medical treatment	18 (16 to 23)	−73·3% (−79·3 to −57·6)	5 (3 to 8)	64·2% (56·2 to 72·3)	23 (20 to 28)	−67·2% (−74·6 to −49·2)
	Animal contact	6 (5 to 8)	−83·1% (−86·6 to −55·1)	5 (3 to 7)	−42·7% (−45·9 to −40·0)	11 (9 to 14)	−75·3% (−80·1 to −50·9)
	Foreign body	90 (78 to 99)	−58·8% (−67·6 to −52·1)	12 (8 to 16)	−17·2% (−23·6 to −9·6)	101 (88 to 111)	−56·2% (−65·3 to −49·8)
	Environmental heat and cold exposure	8 (5 to 10)	−83·6% (−86·3 to −73·5)	15 (10 to 21)	−28·6% (−34·0 to −23·2)	23 (17 to 29)	−67·1% (−73·8 to −53·4)
	Exposure to forces of nature	2 (2 to 2)	−67·4% (−67·4 to −67·4)	2 (2 to 3)	188·0% (143·9 to 235·7)	4 (4 to 5)	−36·4% (−42·9 to −28·3)
	Other unintentional injuries	57 (54 to 60)	−14·8% (−20·0 to −7·5)	11 (8 to 16)	−12·5% (−18·8 to −6·3)	68 (63 to 73)	−14·4% (−18·9 to −8·2)
	**Self-harm and interpersonal violence**	**297 (280 to 335)**	**−72·7% (−75·3 to −64·8)**	**102 (77 to 133)**	**−6·9% (−11·4 to −3·2)**	**399 (367 to 442)**	**−66·7% (−69·9 to −58·9)**
	Self-harm	243 (229 to 273)	−71·9% (−74·4 to −64·7)	2 (2 to 3)	−52·7% (−57·9 to −46·9)	246 (232 to 275)	−71·8% (−74·3 to −64·7)
	Interpersonal violence	51 (46 to 71)	−74·6% (−78·5 to −63·8)	97 (73 to 128)	−4·4% (−8·5 to −1·1)	148 (122 to 184)	−51·0% (−57·6 to −40·7)
	Conflict and terrorism	0 (0 to 0)	−100·0% (−100·0 to −100·0)	0 (0 to 0)	−12·0% (−25·7 to 2·3)	0 (0 to 0)	−45·5% (−56·6 to −31·6)
	Executions and police conflict	3 (2 to 3)	−87·4% (−89·6 to −56·6)	2 (1 to 3)	−15·3% (−29·4 to 16·4)	5 (4 to 6)	−79·7% (−83·3 to −47·3)

Data in parentheses are 95% uncertainty intervals. YLL=year of life lost. YLD=year lived with disability. DALY=disability-adjusted life-year.

**Figure 1:**
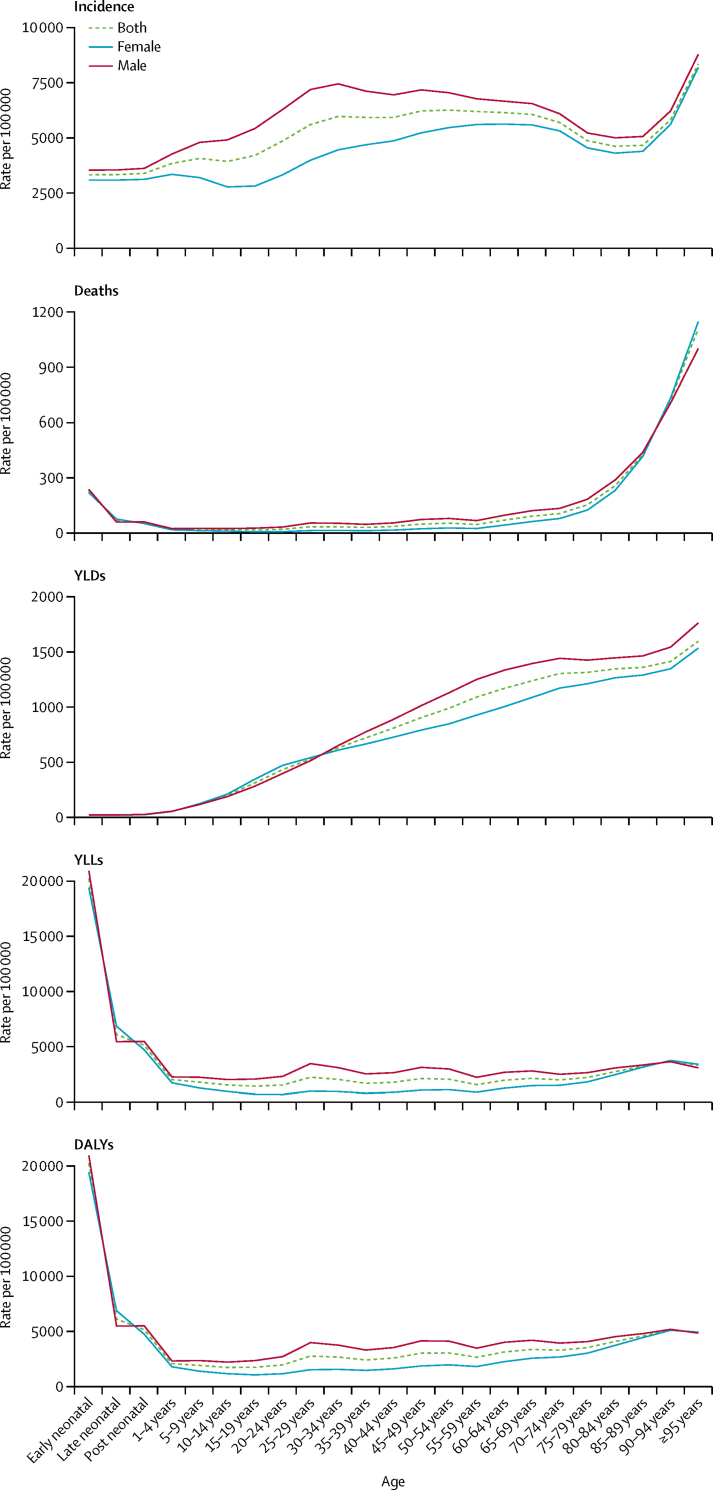
Incidence, death, YLD, YLL, and DALY rates for all injuries by sex and age in China, 2017 YLD=year lived with disability. YLL=year of life lost. DALY=disability-adjusted life-year.

The age curves of incidence saw a gradual increase from early neonatal to 50–54 years of age for both sexes (30–34 years of age in males and 65–69 years of age in females), followed by a decline until 85–89 years of age, with a surge in incidence up to 95 years or older (figure 1). Death rates were relatively high in the first year of life, declining to low values from 1 to 74 years of age, followed by a steep rise from over the age of 75 years (figure 1). The age pattern of DALYs was much flatter than that of deaths after the first year of life because the YLL component of DALYs gives greater weight to deaths occurring in younger individuals and, to a lesser extent, the accumulation of cases with long-term consequences of the more severe injuries (figure 1). The age-standardised incidence, mortality, and DALY rates of all injuries combined were higher in males than in females (figure 1).

Across provinces, age-standardised incidence rates of all injuries varied from a low of 3152 (95% UI 2972–3340) per 100 000 in Gansu to a high of 9907 (9307–10 468) in Beijing (table 3). The age-standardised death rate was lowest in Beijing (17 deaths [95% UI 14–19] per 100 000) and highest in Yunnan (74 deaths [63–86] per 100 000). The age-standardised DALY rate of all injuries ranged from 1335 DALYs (1153–1556) per 100 000 in Macao to 3733 DALYs (3256–4259) per 100 000 in Yunnan (table 3).

**Table 3:** Age-standardised incidence, mortality, YLL, YLD, and DALY rates in 2017 and percentage change from 1990 to 2017 for all injuries in China and provinces

	**Incidence**		**Deaths**		**YLLs**		**YLDs**		**DALYs**	
	2017 age-standardised rates per 100 000	Percentage change in age-standardised rates, 1990–2017	2017 age-standardised rates per 100 000	Percentage change in age-standardised rates, 1990–2017	2017 age-standardised rates per 100 000	Percentage change in age-standardised rates, 1990–2017	2017 age-standardised rates per 100 000	Percentage change in age-standardised rates, 1990–2017	2017 age-standardised rates per 100 000	Percentage change in age-standardised rates, 1990–2017
**China**	**5111 (4827 to 5410)**	**50·6% (46·6 to 54·6)**	**46 (43 to 48)**	**−44·3% (−48·9 to −41·1)**	**1893 (1798 to 1963)**	**−55·8% (−58·8 to −53·4)**	**580 (430 to 758)**	**21·2% (14·9 to 27·9)**	**2473 (2298 to 2679)**	**−48·1% (−51·8 to −44·6)**
Anhui	3760 (3548 to 3990)	31·5% (27·3 to 35·8)	51 (43 to 59)	−55·4% (−62·5 to −47·5)	2110 (1820 to 2415)	−64·5% (−69·9 to −58·2)	431 (320 to 564)	1·0% (−4·9 to 7·7)	2541 (2224 to 2872)	−60·1% (−65·4 to −54·1)
Beijing	9907 (9307 to 10 468)	71·0% (65·0 to 76·8)	17 (14 to 19)	−47·9% (−58·6 to −35·7)	617 (520 to 718)	−56·5% (−65·1 to −46·3)	876 (641 to 1163)	48·9% (42·2 to 55·5)	1493 (1234 to 1799)	−25·6% (−37·5 to −14·0)
Chongqing	4032 (3805 to 4269)	24·5% (20·5 to 28·8)	40 (33 to 48)	−55·1% (−64·4 to −43·9)	1667 (1399 to 1978)	−65·6% (−72·5 to −56·6)	466 (347 to 613)	−3·4% (−10·0 to 3·4)	2134 (1824 to 2489)	−60·0% (−67·2 to −51·1)
Fujian	6619 (6232 to 7031)	64·7% (59·1 to 70·1)	49 (40 to 58)	−39·8% (−51·0 to −27·8)	1624 (1382 to 1894)	−57·8% (−64·4 to −49·9)	682 (501 to 906)	25·7% (17·4 to 34·7)	2306 (2001 to 2653)	−47·5% (−54·4 to −40·1)
Gansu	3152 (2972 to 3340)	7·8% (3·4 to 12·0)	52 (45 to 60)	−56·7% (−63·7 to −49·0)	2397 (2065 to 2752)	−63·3% (−69·4 to −56·9)	409 (308 to 532)	−10·8% (−16·6 to −4·4)	2806 (2457 to 3165)	−59·9% (−65·8 to −53·5)
Guangdong	6444 (6002 to 6919)	76·0% (68·5 to 83·5)	31 (27 to 36)	−44·1% (−54·8 to −32·9)	1257 (1089 to 1447)	−53·2% (−61·6 to −43·7)	657 (483 to 870)	35·7% (26·5 to 44·7)	1914 (1663 to 2201)	−39·6% (−48·7 to −30·4)
Guangxi	3449 (3233 to 3702)	19·4% (14·0 to 25·2)	51 (43 to 60)	−36·8% (−47·9 to −23·8)	2195 (1868 to 2592)	−48·1% (−56·9 to −38·1)	407 (305 to 535)	−4·8% (−11·0 to 2·3)	2602 (2255 to 3010)	−44·2% (−52·7 to −35·0)
Guizhou	5070 (4782 to 5367)	37·4% (32·0 to 42·7)	54 (45 to 64)	−50·4% (−60·7 to −38·2)	2207 (1826 to 2666)	−62·1% (−70·5 to −51·8)	634 (476 to 829)	11·4% (4·3 to 19·3)	2841 (2434 to 3319)	−55·6% (−63·8 to −45·5)
Hainan	5835 (5553 to 6136)	32·5% (29·0 to 36·2)	40 (34 to 47)	−55·4% (−63·9 to −44·5)	1712 (1466 to 1995)	−59·5% (−67·5 to −50·1)	606 (447 to 796)	3·6% (−3·5 to 11·5)	2318 (2018 to 2665)	−51·8% (−59·5 to −42·9)
Hebei	4604 (4308 to 4912)	55·9% (50·0 to 62·2)	47 (41 to 54)	−23·3% (−35·3 to −8·9)	2225 (1927 to 2560)	−31·5% (−41·9 to −19·1)	540 (400 to 709)	23·5% (16·2 to 31·3)	2765 (2435 to 3113)	−24·9% (−34·7 to −13·8)
Heilongjiang	3628 (3390 to 3877)	26·7% (22·0 to 32·0)	29 (24 to 35)	−49·4% (−58·4 to −40·1)	1329 (1122 to 1571)	−53·6% (−61·3 to −45·0)	413 (310 to 541)	0·6% (−4·5 to 6·1)	1741 (1512 to 2002)	−46·8% (−53·8 to −38·6)
Henan	3387 (3149 to 3646)	36·9% (30·5 to 43·5)	49 (41 to 57)	−33·4% (−44·6 to −21·7)	2015 (1705 to 2349)	−48·1% (−57·3 to −38·4)	399 (300 to 522)	3·4% (−3·7 to 10·9)	2414 (2093 to 2763)	−43·4% (−52·0 to −34·2)
Hong Kong SAR	6256 (5796 to 6785)	58·8% (53·2 to 64·1)	19 (16 to 22)	−36·9% (−47·4 to −24·8)	678 (570 to 807)	−44·2% (−53·7 to −33·0)	698 (512 to 925)	53·1% (48·5 to 57·3)	1376 (1153 to 1638)	−17·7% (−27·8 to −6·8)
Hubei	4058 (3807 to 4326)	31·6% (26·2 to 37·4)	67 (53 to 78)	−35·5% (−46·0 to −23·8)	2322 (1927 to 2690)	−52·8% (−59·7 to −44·6)	490 (363 to 644)	6·2% (−0·8 to 13·1)	2812 (2391 to 3215)	−47·7% (−54·4 to −39·8)
Hunan	3780 (3561 to 4006)	29·2% (24·7 to 34·2)	57 (48 to 66)	−41·6% (−51·1 to −30·8)	2294 (1964 to 2685)	−53·2% (−60·5 to −44·7)	435 (325 to 569)	−0·7% (−7·4 to 6·9)	2730 (2377 to 3112)	−48·9% (−56·1 to −41·1)
Inner Mongolia	4428 (4144 to 4711)	41·9% (37·0 to 47·1)	38 (32 to 44)	−54·0% (−61·9 to −44·3)	1702 (1468 to 1978)	−59·4% (−66·1 to −51·3)	513 (379 to 676)	8·5% (1·7 to 15·6)	2216 (1933 to 2512)	−52·5% (−59·2 to −45·2)
Jiangsu	8097 (7654 to 8567)	84·5% (78·8 to 90·1)	42 (36 to 49)	−39·4% (−50·0 to −28·6)	1431 (1224 to 1659)	−58·7% (−65·3 to −51·5)	813 (594 to 1085)	46·2% (37·3 to 55·4)	2244 (1975 to 2588)	−44·2% (−51·6 to −35·6)
Jiangxi	5219 (4909 to 5540)	40·2% (35·0 to 45·2)	56 (49 to 64)	−58·2% (−64·7 to −51·0)	2467 (2152 to 2798)	−66·0% (−71·5 to −59·8)	623 (461 to 818)	16·2% (10·2 to 22·3)	3090 (2719 to 3493)	−60·4% (−66·1 to −53·7)
Jilin	3309 (3073 to 3561)	27·5% (21·8 to 33·5)	26 (21 to 33)	−51·8% (−61·4 to −40·4)	1201 (971 to 1479)	−55·5% (−64·3 to −44·0)	422 (319 to 552)	9·0% (4·5 to 13·3)	1623 (1362 to 1907)	−47·4% (−55·4 to −37·1)
Liaoning	4810 (4506 to 5126)	7·5% (3·7 to 11·0)	30 (25 to 36)	−41·6% (−52·6 to −28·0)	1249 (1046 to 1468)	−48·9% (−58·4 to −37·3)	524 (389 to 688)	−6·5% (−9·7 to −3·3)	1773 (1531 to 2040)	−41·0% (−49·6 to −31·3)
Macao SAR	5252 (4916 to 5587)	49·4% (44·9 to 54·0)	20 (18 to 23)	−49·7% (−56·3 to −41·2)	735 (652 to 848)	−54·1% (−60·1 to −46·8)	600 (443 to 790)	40·3% (35·6 to 44·5)	1335 (1153 to 1556)	−34·2% (−41·5 to −26·2)
Ningxia	3492 (3257 to 3752)	8·6% (4·2 to 13·9)	53 (45 to 64)	−49·1% (−59·1 to −35·8)	2439 (2068 to 2882)	−60·1% (−68·8 to −48·9)	449 (339 to 585)	−3·5% (−7·6 to 0·5)	2887 (2485 to 3351)	−56·1% (−64·6 to −45·4)
Qinghai	3332 (3079 to 3601)	9·3% (3·7 to 15·2)	61 (52 to 71)	−45·4% (−56·3 to −32·5)	2859 (2442 to 3378)	−54·3% (−63·7 to −42·6)	471 (358 to 607)	6·2% (2·3 to 10·1)	3330 (2908 to 3883)	−50·3% (−59·6 to −38·9)
Shaanxi	3842 (3605 to 4081)	37·9% (32·4 to 43·7)	47 (38 to 55)	−13·3% (−29·6 to 6·6)	2062 (1713 to 2437)	−31·6% (−44·2 to −16·0)	494 (370 to 645)	10·2% (4·3 to 15·9)	2555 (2170 to 2943)	−26·2% (−37·5 to −12·4)
Shandong	4194 (3962 to 4434)	40·0% (35·3 to 44·9)	40 (34 to 48)	−52·2% (−60·7 to −41·8)	1659 (1407 to 1952)	−58·4% (−65·7 to −49·6)	500 (371 to 654)	20·6% (15·3 to 25·9)	2159 (1873 to 2483)	−50·9% (−58·4 to −42·4)
Shanghai	9869 (9178 to 10 639)	88·6% (80·8 to 96·9)	24 (21 to 28)	−38·3% (−49·9 to −24·7)	966 (815 to 1126)	−47·7% (−56·9 to −36·4)	1105 (811 to 1468)	75·8% (69·5 to 82·0)	2071 (1732 to 2458)	−16·3% (−28·6 to −3·1)
Shanxi	5287 (4991 to 5596)	53·9% (49·2 to 59·2)	38 (32 to 46)	−43·9% (−55·5 to −31·2)	1702 (1433 to 1999)	−50·4% (−60·3 to −39·8)	618 (458 to 812)	31·9% (26·2 to 37·3)	2320 (1986 to 2657)	−40·5% (−50·8 to −30·3)
Sichuan	5913 (5566 to 6305)	65·9% (59·7 to 71·8)	52 (43 to 61)	−39·7% (−51·7 to −27·0)	2184 (1841 to 2598)	−56·7% (−65·8 to −45·5)	687 (517 to 903)	35·7% (27·8 to 43·5)	2871 (2456 to 3339)	−48·2% (−57·7 to −37·2)
Taiwan	4479 (4239 to 4738)	8·0% (3·7 to 12·1)	40 (39 to 43)	−54·2% (−56·5 to −51·8)	1580 (1497 to 1667)	−59·8% (−61·9 to −57·5)	508 (373 to 669)	−1·9% (−5·0 to 1·1)	2088 (1936 to 2260)	−53·1% (−55·6 to −50·4)
Tianjin	3740 (3499 to 4002)	32·6% (27·5 to 38·1)	31 (26 to 36)	−36·6% (−47·7 to −22·8)	1373 (1182 to 1599)	−41·8% (−51·1 to −29·6)	456 (342 to 596)	19·9% (15·4 to 23·9)	1830 (1598 to 2068)	−33·2% (−42·8 to −22·3)
Tibet	3784 (3497 to 4092)	5·3% (0·4 to 10·2)	56 (47 to 65)	−52·9% (−61·2 to −42·9)	2656 (2243 to 3147)	−56·6% (−65·6 to −45·7)	519 (397 to 667)	14·2% (10·8 to 17·1)	3175 (2731 to 3679)	−51·7% (−60·6 to −41·2)
Xinjiang	3375 (3178 to 3597)	32·3% (27·6 to 37·7)	51 (43 to 59)	−36·7% (−47·6 to −21·9)	2588 (2227 to 2988)	−43·6% (−54·5 to −28·8)	451 (343 to 582)	11·6% (6·4 to 16·4)	3039 (2662 to 3451)	−39·2% (−49·8 to −24·8)
Yunnan	4740 (4480 to 5030)	24·5% (19·5 to 29·3)	74 (63 to 86)	−44·4% (−55·2 to −32·6)	3162 (2718 to 3641)	−56·4% (−64·8 to −46·7)	571 (430 to 740)	8·7% (3·4 to 13·7)	3733 (3256 to 4259)	−52·0% (−60·2 to −42·4)
Zhejiang	8566 (8093 to 9062)	73·2% (67·6 to 79·0)	47 (38 to 56)	−47·2% (−56·1 to −36·2)	1531 (1306 to 1800)	−63·6% (−68·9 to −56·5)	943 (691 to 1243)	49·9% (43·4 to 55·9)	2474 (2130 to 2859)	−48·9% (−55·4 to −41·6)

Data in parentheses are 95% uncertainty intervals. YLL=year of life lost. YLD=year lived with disability. DALY=disability-adjusted life-year. SAR=special administrative region.

**Figure 2:**
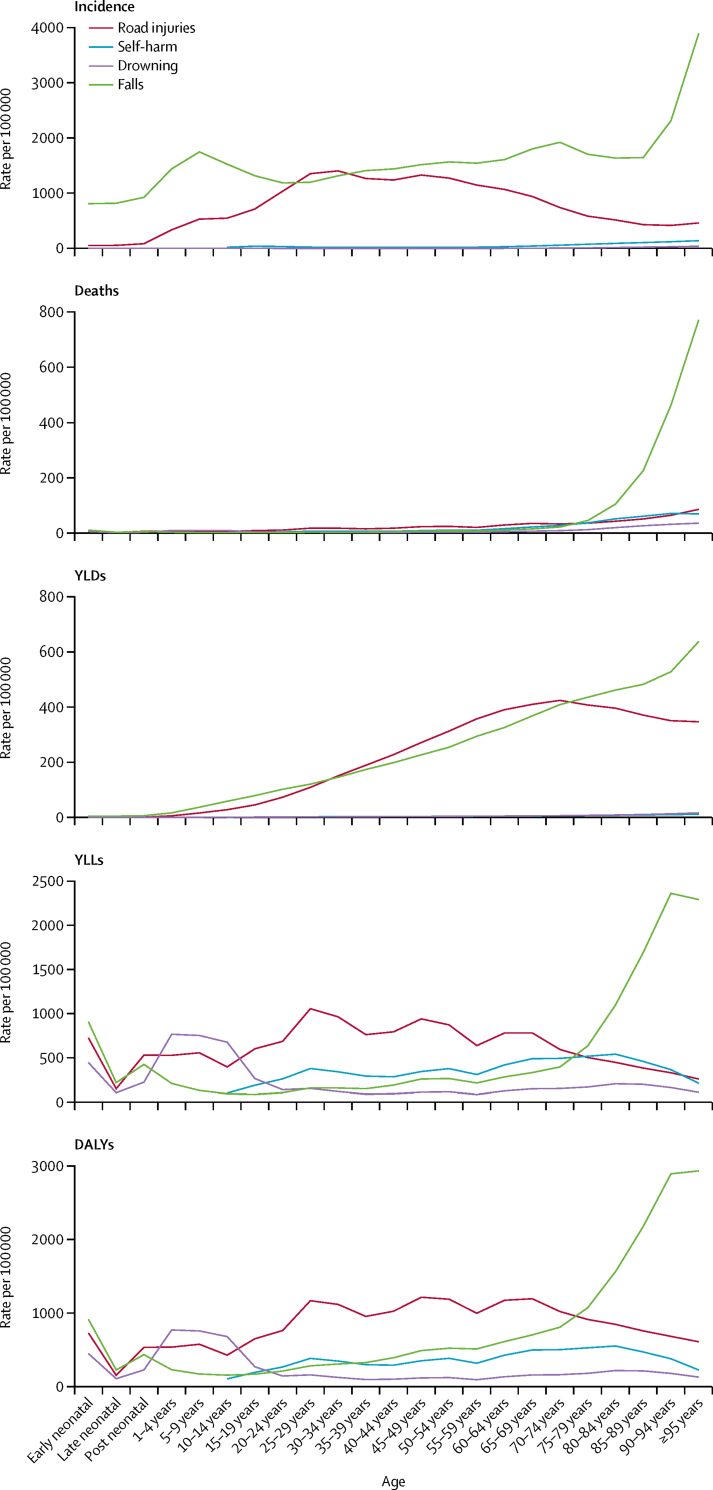
Age-specific incidence, death, YLD, YLL, and DALY rates by cause of injury in China, 2017 Self-harm is measured from age 10–14 years onwards. YLD=year lived with disability. YLL=year of life lost. DALY=disability-adjusted life-year.

Among different causes of injury in China, the four level 3 injuries with the highest age-standardised DALY rates were road injuries, falls, drowning, and self-harm, each with a distinct age pattern (figure 2; table 2). Road injuries peaked in early adulthood, were relatively stable between age ranges 25–29 and 60–69 years, and fell in the later years of life. Drowning peaked in childhood, whereas self-harm rates showed a small peak in the 25–29 years and 80–84 years age groups (figure 2). In terms of the geographical pattern of the four leading causes of age-standardised DALYs, there was a large disparity in road injuries between eastern and western districts, with the lowest rate of 294 DALYs (95% UI 244–358) per 100 000 in Hong Kong and highest rate of 1343 DALYs (1151–1615) per 100 000 in the Qinghai (appendix p 6). The age-standardised DALY rate of falls ranged from 153 DALYs (114–221) per 100 000 in Jilin to 662 DALYs (441–812) per 100 000 in Yunnan (appendix p 7). For drowning, the difference in age-standardised DALYs ranged from 51 DALYs (42–65) per 100 000 in Beijing to 544 DALYs (445–654) per 100 000 in Xinjiang (appendix p 8). The highest rate of age-standardised DALYs for self-harm was in Taiwan with 542 DALYs (509–578) per 100 000 and the lowest was in Beijing with 104 DALYs (81–131) per 100 000 (appendix p 9).

The age-standardised incidence rate of all injuries increased by 50·6% (95% UI 46·6–54·6) at the national level between 1990 and 2017 (table 1). Despite the increase in age-standardised incidence, China experienced a substantial decrease in age-standardised mortality rate (44·3% decrease, 95% UI 41·1–48·9) and age-standardised DALY rates (48·1%, 44·6–51·8) for all injuries between 1990 and 2017 (Table 1, Table 2). Age-standardised DALY rates for drowning; injuries from fire, heat, and hot substances; adverse effects of medical treatments; animal contact; environmental heat and cold exposure; self-harm; and executions and police conflict declined by more than 60% between 1990 and 2017 (table 2).

The age-standardised incidence rate of road injuries in 2017 more than doubled, increasing by 113·2% (95% UI 103·7 to 123·6) from 1990 to 2017 (table 1). However, age-standardised mortality and DALY rates from road injuries decreased by 21·8% (14·9 to 30·7) and 25·0% (18·8 to 32·5), respectively (Table 1, Table 2). Age-standardised DALY rates of road injuries varied from an increase of 9·5% (−10·8 to 31·9) in Shaanxi to a decrease of 63·5% (60·6 to 66·0) in Taiwan (appendix p 6).

The age-standardised incidence rate of falls increased by 113·2% (95% UI 104·4 to 123·6) between 1990 and 2017 (table 1). The age-standardised mortality rate for falls increased by 12·8% (−23·9 to 33·4), whereas the DALY rate showed no substantial change over this time period (Table 1, Table 2). Age-standardised DALY rates from falls decreased in 16 provinces but increased in 18 others (appendix p 7). Tibet experienced the largest decrease (40·4%, 95% UI 22·5 to 53·2) whereas the largest increase occurred in Shanghai (46·8%, 24·3 to 66·7; appendix p 7).

The age-standardised incidence rate of drowning decreased by 17·4% (95% UI 10·2–23·8) from 1990 to 2017 (table 1). Age-standardised mortality and DALY rates decreased by 65·3% (62·9–67·5) and 71·3% (69·1–73·3), respectively (Table 1, Table 2). Age-standardised DALY rates decreased in all provinces, with decreases varying from 46·9% (35·8–56·2) in Hong Kong to 80·2% (78·4–81·8) in Taiwan (appendix p 8).

The age-standardised incidence rate of self-harm decreased by 39·4% (95% UI 33·8–45·1) from 1990 to 2017, while age-standardised mortality and DALY rates of self-harm decreased by 65·6% (95% UI 57·6–68·7) and 71·8% (64·7–74·3), respectively (Table 1, Table 2). Taiwan had a 43·9% (95% UI 34·7–54·8) increase in age-standardised DALY rates, whereas 33 provinces had decreases varying from 41·1% (27·9–52·3) in Hong Kong to 82·9% (76·4–86·6) in Jiangxi (appendix p 9).

**Figure 3:**
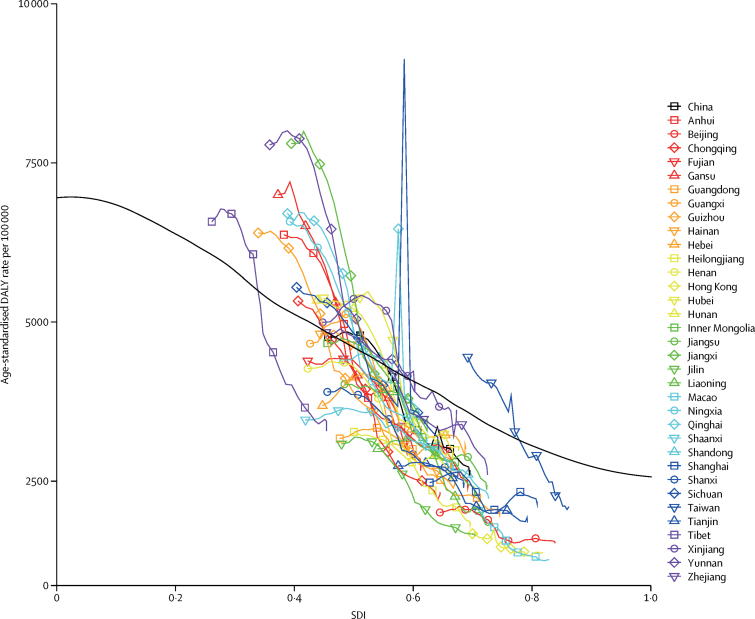
Age-standardised DALYs by province and SDI value The black line shows the expected pattern of injury DALY rates with sociodemographic development based on all GBD locations. DALY=disability-adjusted life-year. SDI=Socio-demographic Index.

Relative to the expected pattern of injury DALY rates with sociodemographic development based on all GBD locations, provinces in China have shown faster decline as their SDI has increased (figure 3). In other words, between 1990 and 2017, China achieved a greater reduction in age-standardised DALY rates from injuries than the global average. Note that the sharp interruptions in the DALY rates for several provinces were due to natural disasters (eg, earthquakes in Sichuan in 2008 and in Qinghai in 2010).

## Discussion

This study on the burden of injuries in China revealed that although the incidence of injuries increased between 1990 and 2017, the injury burden in terms of mortality and DALYs greatly improved. This change is not surprising for China, which has experienced a rapid social and economic development over the past three decades. On the one hand, transformation of the social economy has accelerated the process of motorisation and industrialisation, leading to the arrival of motorised society and environmental pollution that has dramatically increased the possibility of road injuries and has been reported as a risk factor for fall-related injury.^15^, ^16^, ^17^ On the other hand, the burden of fatal injury outcomes might have been reduced by the substantial socioeconomic and political developments that have occurred in the past 30 years.^5^, ^15^ Of these developments, the most important were probably reduced poverty, higher employment, availability and coverage of medical and basic public health services, formulation of injury prevention-related policies and legislations, active government-supported initiatives, and increasing the injury prevention awareness of the population, particularly for child injury prevention.^4^, ^5^ Child protection strategies have been widely acknowledged and publicly supported, and there has been some speculation that the one-child policy has led to greater parental attention to children, thus driving large improvements in younger age groups, given that many child injuries might be due to lack of supervision or unsafe circumstances.^5^, ^18^

Economic development has a complex relationship with road injuries, as it has been reported that road injury death increases at the early stage of economic development.^19^, ^20^, ^21^ In low-income countries, car crashes and road injury deaths have been estimated to increase by 4·7% and 3·1%, respectively, for each 10% increase in gross domestic product (GDP).^19^, ^20^, ^21^ In China, GDP has increased by 30 times over the past two decades, with increases of highway mileage from 1·03 to 4·36 million km and of motor vehicle users from 14·76 million to 25·01 million, contributing to an increased probability of road injury.^22^, ^23^, ^24^ However, improvements in road construction, expansion of road safety awareness, and increased motor vehicle safety systems have occurred in parallel with these economic developments, which might explain why increases in incidence were accompanied by decreases in mortality.^25^ Road safety laws are likely to have played a role. The Regulation of Road Traffic Management was formulated in 1988 and included requirements to use seatbelts and helmets. The Road Traffic Safety Law was then enacted in 2003 and included more stringent road traffic safety regulations pertaining to drinking and driving, speeding, lane use, and the licencing system.^26^, ^27^ In addition, an inter-ministry project involving 27 government departments was established in 2003 with the purpose of improving road safety.^28^ Additionally, in 2011, drinking and driving and speeding became offences under criminal law in China.^29^ Finally, regulations targeted at road safety in the vicinity of schools, championed by ten ministries, might have contributed to the decline in burden of road injury in children.^30^

The burden of self-harm also greatly declined during the study period. One of the key strategies of suicide prevention has been to restrict access to the means of suicide.^31^, ^32^ The Chinese Government enforced a law in 1981 to strictly control the manufacturing, sales, and use of firearms.^33^ Regulations enacted in 1997 and revised in 2001 also increased the supervision and management of production, marketing, and use of pesticides and other agricultural chemicals.^34^ In 2005, a regulation was enacted to reinforce the management of narcotic and psychotropic drugs to improve the safety of using these drugs.^35^ Moreover, the prevention of self-harm might have benefited from efforts to improve mental health services, given that a large proportion of individuals who die by suicide in China have a mental illness.^36^ The National Mental Health Work Plan (2015–2020), jointly issued by the National Health and Family Planning Commission, the Ministry of Civil Affairs, the Ministry of Public Security, and the China Disabled Persons’ Federation, proposed a goal of improving mental health services by strengthening advocacy for mental health issues and reinforcing interventions for populations with psychological and behavioural issues.^37^ The first national mental health law, implemented in 2013, included treatment standards and treatment access protections^38^ that led to 4·3 million people with mental health issues being registered for services, among whom 73·2% had received services by the end of 2014.^37^, ^39^ The decline in suicide in females might be associated with improvements in economic equality, such as increased educational opportunities and freedom of marriage. Despite the overall improvements in the burden of self-harm, older people (ie, aged ≥60 years) continue to experience considerable burden. Older people have been found to be susceptible to self-harm risk factors such as isolation and barriers to accessing health care, particularly for the so-called empty nest elderly,^32^, ^40^, ^41^ who might be prone to depression, sadness, and grief after the departure of their children and who can face substantial financial pressure when coping with chronic diseases after retirement.^42^, ^43^

The burden of drowning also greatly decreased between 1990 and 2017. Since 2007, the Ministry of Education has disseminated annual warnings about the risks of drowning and advises adult supervision near bodies of water.^44^ This increased awareness might have partly mitigated the risk of drowning. Additionally, the enrolment rate for kindergarten has increased in the past 15 years,^45^ which has probably decreased the amount of exposure young children have to bodies of water. Improved infrastructure projects including bridges, roads, levees, safe drinking water, and sanitary washroom programmes are likely to have also decreased exposure to bodies of water.^46^ Improved waterborne traffic safety regulations have also been legislated in the past few decades, which might play a role.^47^

The burden of falls declined overall from 1990 to 2017, but with a notable increase in burden among older people. This is probably explained by the 8·5-year increase in life expectancy during the past two decades, resulting in a greater proportion of older people living with chronic diseases at risk of fall-related injuries and death.^48^ As discussed previously, the older population living without familial or social support might be at higher risk of disability or death due to delayed care and treatment for fall-related injuries.^40^, ^42^, ^49^ Another possible factor responsible for the high burden of falls among older people could be the long-term exposure to ambient particulate matter, which has been reported as an important risk factor of fall-related injury.^17^ Given the high burden of falls in older people, a large economic burden of falls and fall-related injury could be reasonably anticipated, implying that more attention should be given to this looming health crisis, especially to achieve the sustainable development goals of healthy ageing.^50^

This research, as a part of the GBD study, included the same limitations previously discussed in GBD 2017 literature, particularly as they pertained to burden estimation in China.^6^, ^7^, ^8^, ^9^, ^10^, ^11^ One of the main limitations of measuring injury burden in China was a lack of reliable injury incidence data. Although the NISS^13^ and the China Zhuhai injury patient follow-up study^14^ are important sources of injury data in China, these data are not nationally representative and data from before 2006 were unavailable. Data on injuries before 2006 could only be derived from sparse existing literature sources, which caused the modelling process for injuries to depend more heavily on cause of death estimates, covariates, and data from surrounding countries. The mortality component in terms of YLLs constituted close to 80% of injury DALYs in China and therefore DALY estimates were most sensitive to the analyses of cause of injury death data. In the 1990s and part of the 2000s, cause of death estimates were available for a sample of the country only. However, these data were considered to be representative for the provinces. It is possible that some part of the large declining trend was influenced by the lesser coverage and quality of cause of death data in the 1990s, but the declines recorded were so large that it is unlikely the trends we report are spurious. Moreover, data from the sample registration system in recent years aligned well with the vital registration data that achieved large increases in coverage over the past decade,^8^ which could also avoid the underestimation of the burden of road injuries caused by policy-reported data.^51^

Furthermore, as the DSP system and the NISS are the primary data sources for fatal and non-fatal injury analysis in China, the accuracy of results presented here is certainly subject to the quality and reliability of the input data from these surveillance systems. As with all vital registration and surveillance systems, issues such as misclassification and under-reporting can substantially affect outcomes based on such data. However, strict internal data quality auditing methods such as the under-reporting survey done for the DSP system and other reviewing procedures at different levels of CDCs (county, precinct, provincial, and national), including cross-checking using multiple sources based on the electronic surveillance system management platforms, have been implemented to continuously improve the quality of the data from the two surveillance systems. To further improve the quality of the data from these systems, additional efforts need to be made. First, the existing training, implementation, and assessment of data quality control measures should be reinforced for all staff at all levels of institutions involved in the data collection processes of these two systems. Second, data sharing among researchers, both domestic and international, should be encouraged. Improved use of data can further help to improve the quality of data in the future. Third, more efforts could be made to adopt more advanced information technology and big data methods to bridge the gap of the available data and the best information on population health in China.

In conclusion, although the overall incidence of injuries in China has increased since 1990, the burden in terms of DALYs and cause-specific mortality has decreased, particularly among the key causes of injury, including road injury, self-harm, drowning, and falls. These patterns were probably due to the complex socioeconomic and demographic changes during this time period, which probably led to a greater number of individuals being exposed to risks such as motor vehicle accidents and falls but also led to infrastructure and health-care improvements. The trends described in this study might be useful in guiding future policy development and health-care system investments that aim to both decrease the incidence of injuries and increase the access to medical care when injuries do occur. These estimates and trends might also depict what other emerging economies should anticipate as they progress through the epidemiological transition.

## Data sharing

Additional results are available on the IHME website.
